# Comprehensive functional profiling of long non-coding RNAs through a novel pan-cancer integration approach and modular analysis of their protein-coding gene association networks

**DOI:** 10.1186/s12864-019-5850-7

**Published:** 2019-06-03

**Authors:** Kevin Walters, Radmir Sarsenov, Wen Siong Too, Roseanna K. Hare, Ian C. Paterson, Daniel W. Lambert, Stephen Brown, James R. Bradford

**Affiliations:** 1 School of Mathematics and Statistics, University of Sheffield, Sheffield, South Yorkshire UK; 2 Sheffield RNAi Screening Facility (SRSF), Department of Biomedical Science, University of Sheffield, Sheffield, South Yorkshire UK; 3 Department of Biomedical Science, University of Sheffield, Sheffield, South Yorkshire UK; 4 Department of Oral and Craniofacial Sciences, Faculty of Dentistry, University of Malaya, Kuala Lumpur, Malaysia; 5 Sheffield Institute for Nucleic Acids (SInFoNiA), Integrated Biosciences, School of Clinical Dentistry, University of Sheffield, Sheffield, South Yorkshire UK; 6 Sheffield Institute for Nucleic Acids (SInFoNiA), Department of Oncology and Metabolism, University of Sheffield, Sheffield, South Yorkshire UK; 7 Almac Diagnostic Services, Craigavon, Northern Ireland, UK

**Keywords:** lncRNA, Functional profiling, Genes networks, Cancer, Epithelial-mesenchymal transition, Extracellular matrix, Tumour microenvironment

## Abstract

**Background:**

Long non-coding RNAs (lncRNAs) are emerging as crucial regulators of cellular processes in diseases such as cancer, although the functions of most remain poorly understood. To address this, here we apply a novel strategy to integrate gene expression profiles across 32 cancer types, and cluster human lncRNAs based on their pan-cancer protein-coding gene associations. By doing so, we derive 16 lncRNA modules whose unique properties allow simultaneous inference of function, disease specificity and regulation for over 800 lncRNAs.

**Results:**

Remarkably, modules could be grouped into just four functional themes: transcription regulation, immunological, extracellular, and neurological, with module generation frequently driven by lncRNA tissue specificity. Notably, three modules associated with the extracellular matrix represented potential networks of lncRNAs regulating key events in tumour progression. These included a tumour-specific signature of 33 lncRNAs that may play a role in inducing epithelial-mesenchymal transition through modulation of TGFβ signalling, and two stromal-specific modules comprising 26 lncRNAs linked to a tumour suppressive microenvironment and 12 lncRNAs related to cancer-associated fibroblasts. One member of the 12-lncRNA signature was experimentally supported by siRNA knockdown, which resulted in attenuated differentiation of quiescent fibroblasts to a cancer-associated phenotype.

**Conclusions:**

Overall, the study provides a unique pan-cancer perspective on the lncRNA functional landscape, acting as a global source of novel hypotheses on lncRNA contribution to tumour progression.

**Electronic supplementary material:**

The online version of this article (10.1186/s12864-019-5850-7) contains supplementary material, which is available to authorized users.

## Background

The advent of high-throughput genomic technologies such as Next Generation Sequencing (NGS) has led to remarkable progress over the last decade in detecting novel transcripts, many of which have no apparent protein-coding capacity. A significant proportion of these non-coding species are long non-coding RNAs (lncRNAs), which typically exceed 200 nucleotides in length, and function through a variety of mechanisms including remodelling of chromatin, transcriptional co-activation/repression, protein inhibition, post-transcriptional modification, or decoy. They are now emerging as crucial regulators of cellular processes and diseases, and their aberrant transcription can lead to altered expression of several important target genes involved in cancer [1], resulting in tumour progression and poor prognosis [2–6].

Despite advances, the vast majority of lncRNAs identified through large-scale efforts such as GENCODE [7] and MiTranscriptome [8] remain poorly understood. To address this gap, several computational approaches have been developed with the ability to assign putative function to thousands of lncRNAs simultaneously by exploiting the widespread availability of cancer genomic data [9, 10]. These methods typically employ a “guilt-by-association” strategy, deriving a prediction based on a common expression pattern between the lncRNA and a biological process or pathway [11]. More recent efforts attempt to strengthen predictions by combining transcriptomic data across multiple cancer [12–15], or normal tissue types [16]. However, whilst representing important advances, these have so far employed limited integration strategies, either seeking consensus across separate lncRNA signatures derived from a small number of cancer types [12, 13], correlation across a single dataset against a restricted set of cancer genes [15], or focusing on a natural antisense transcripts only [16].

To address these shortcomings, we have developed a unique workflow to integrate expression associations between lncRNA and protein coding (PC) genes across 32 different cancer types from The Cancer Genome Atlas (TCGA) to provide a more robust lncRNA-PC association network than can be derived from any single cancer type alone. The workflow incorporates three novel aspects: (1) An Expectation Maximisation (EM) algorithm for estimating the correlation between a lncRNA and PC gene that specifically addresses low lncRNA expression relative to PC gene expression. (2) A statistical method for integrating lncRNA-PC correlations across multiple cancer types to derive a single multi-cancer association (MCA) score between each lncRNA and PC gene, allowing subsequent construction of a single pan-cancer lncRNA-PC gene network. (3) A unique application of Weighted Gene Correlation Network Analysis (WGCNA) [17] to the lncRNA-PC MCA score network allowing its de-convolution into lncRNAs that share consistently similar expression profiles across multiple cancers, henceforth termed “modules”.

Through detailed characterisation of these modules, we provide the most comprehensive pan-cancer assessment of lncRNA-PC gene expression associations to date, allowing simultaneous hypothesis generation on lncRNA function, disease specificity, and transcription factor regulation. More specifically, the unique global perspective of our modular approach reveals the potential for both coordinated and antagonistic lncRNA expression to underpin disease pathway regulation, and new insights into the role of lncRNAs in the tumour microenvironment.

## Results and discussion

### A workflow to identify lncRNA modules based on their pan-cancer protein coding gene associations

**Fig. 1:**
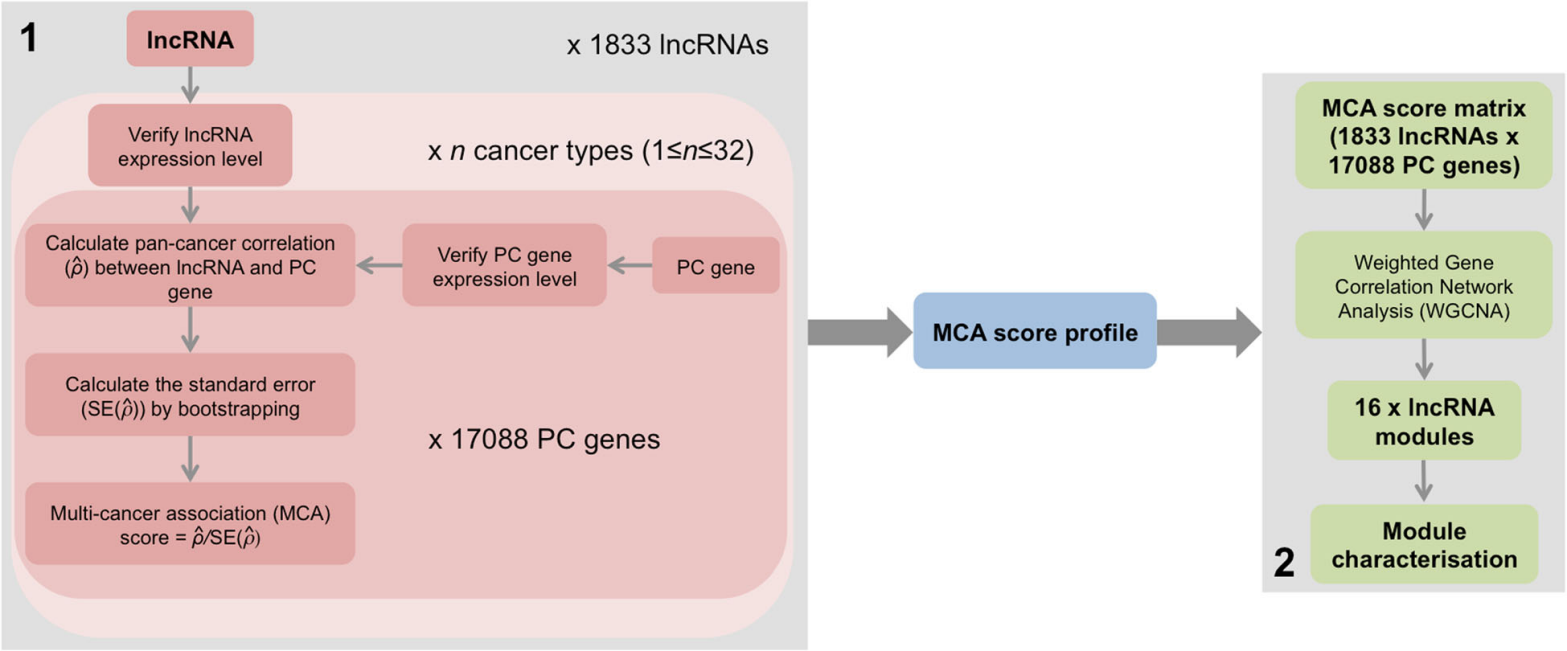
Schematic of workflow to identify lncRNA modules based on their pan-cancer PC gene associations. (1) Derivation of a multi-cancer association (MCA) score profile for 1833 lncRNAs that pass expression level criteria, and (2) identification of 16 modules of highly correlated lncRNAs using Weighted Gene Correlation Analysis (WGCNA) and a matrix of 1833 lncRNA MCA score profiles across 17,088 PC genes as input

The workflow is divided into two main stages (Fig. 1). In the first stage, RNA-Seq expression estimates of each lncRNA and PC gene annotated by GENCODE [7] were inspected across all 32 cancer types (Additional file 4: Table S1), and those that failed to achieve sufficient expression signal in any cancer type were removed (see *Methods* for specific criteria). 1833 lncRNAs expressed in at least one cancer type remained after filtering. An EM algorithm was then applied to estimate a pan-cancer correlation coefficient ($\hat{\rho}$) between the expression profiles of each of these 1833 lncRNAs and 17,088 PC genes across 1 ≤ *n* ≤ 32 cancer types in which the lncRNA expression threshold had been met. The approach was specifically developed to handle instances where lncRNA expression is either low, absent, or undetectable across an excessive number of samples, even if initial expression level criteria had been met. Bootstrapping then quantified the uncertainty of $\hat{\rho}$ in the form of a standard error (SE($\hat{\rho}$)), from which a multi-cancer association (MCA) score was derived: MCA= $\hat{\rho}/ SE\left(\hat{\rho}\right)$. The MCA score calculation was repeated across all PC genes to generate an MCA profile of 17,088 scores for each of the 1833 lncRNAs. The lncRNA-PC gene combination achieving the highest MCA score represented the strongest pan-cancer expression association for that lncRNA. Collectively the profiles formed a matrix of 1833 × 17088 MCA scores. Full details of the derivation of $\hat{\rho}$ and SE($\hat{\rho}$) are described in *Methods*.

**Fig. 2:**
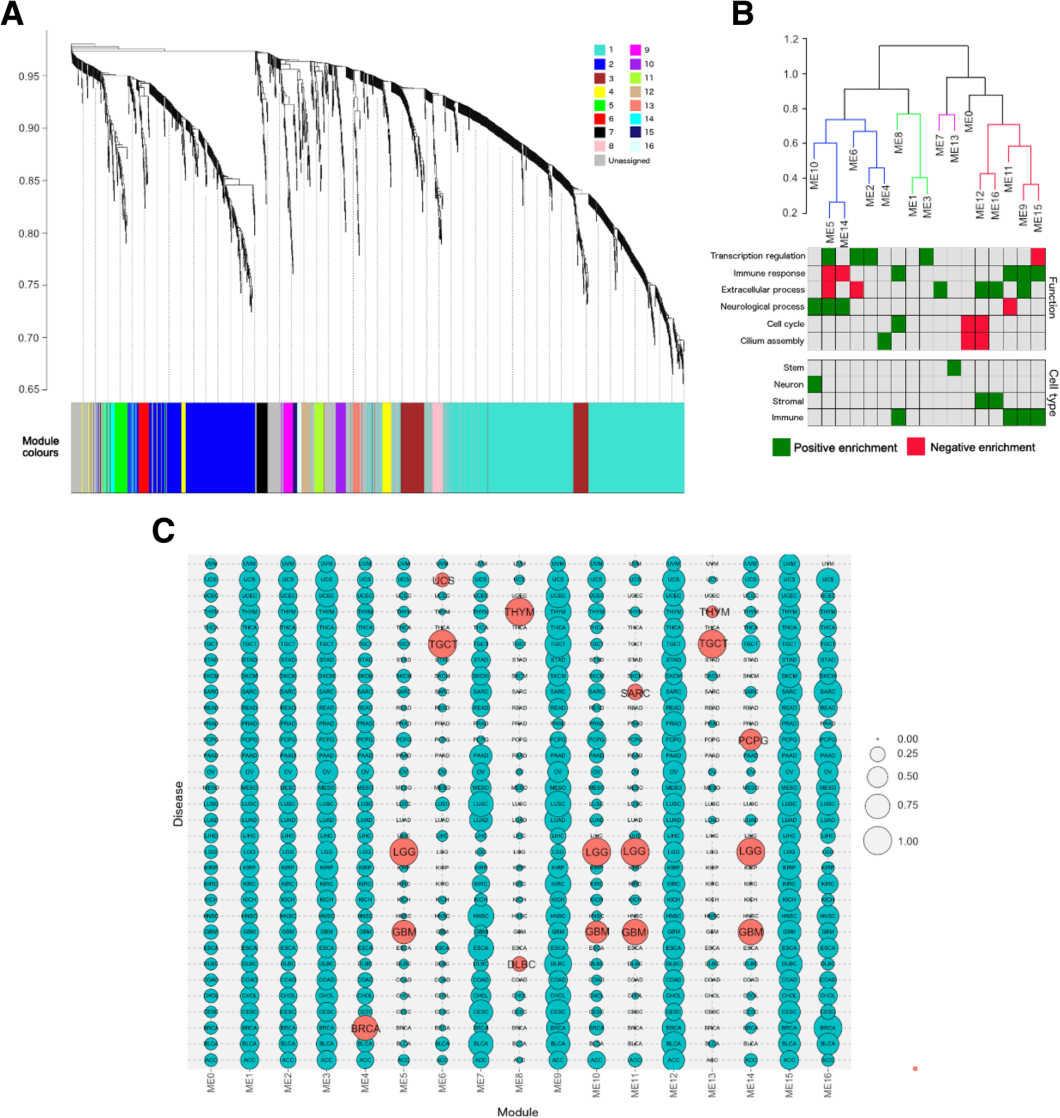
Module characterisation. **a** Dendrogram showing hierarchical clustering of lncRNAs based on MCA score profile. Branches of the dendrogram correspond to modules, with lncRNAs in each module assigned the same colour (indicated by the colour band below the dendrogram). LncRNAs not assigned to a module are coloured grey. **b** Clustering dendrograms of module eigen-lncs. Meta-modules are defined at height cut-off of 0.80 and indicated by different colours. Below the dendrogram, functional and cell type signatures of each module are indicated, with green corresponding to significant positive enrichment and red to significant negative enrichment. **c**. Bubble chart showing cancer type specificity of each module. Size of bubble indicates the proportion of module-associated lncRNAs that meet the expression detection threshold in each cancer type. Red bubbles indicate outlier cancer types (> 1.5 times the interquartile range above the upper quartile). A description of the cancer type codes is given in Additional file 4: Table S1

In stage two, we applied WGCNA [17] to the MCA score matrix. WGCNA is often used as a dimensionally reduction method in genomics, typically applied to gene expression networks of several thousand genes to identify a small number of modules of related genes whose expression profiles are highly correlated. Each module is represented by an eigen-gene, which can be used to correlate modules with meta-data such as clinical traits. The correlation of a gene’s expression profile with a module eigen-gene (ME) provides a measure of significance of the relationship between gene and module. Here, we adapted WGCNA to generate “eigen-lncs”, which are analogous to eigen-genes, to identify 16 modules of lncRNAs with highly correlated MCA score profiles (Fig. 2a; Additional file 5: Table S2). An important advantage of this approach is that the eigen-lnc coefficients attributed to each PC gene (henceforth referred to PC-module association or PC-MA values) can be used as a surrogate for the strength of the relationship between PC gene and eigen-lnc (Additional file 6: Table S3). This allowed for functional traits representative of each module to be identified since each module is related to a set of highly annotated PC genes. Here, we defined PC genes achieving PC-MA > 0.02 as “pro-module” (PC genes whose mRNA expression is consistently positively correlated with members of the module), and PC-MA < -0.02 as “anti-module” (PC genes consistently negatively correlated with the module).

### Module characterisation

#### Common functional traits

Functional enrichment analysis of pro-module PC genes revealed striking properties of lncRNA-PC gene associations (Table 1, Fig. 2b, Additional file 7: Table S4). Primarily, modules could be grouped into four functional signatures: immune, extracellular, transcription regulation, and neurological, broadly corresponding to four sets of positively correlated eigen-lncs or “meta-modules” (Fig. 2b, Additional file 1: Figure S1). Only ME4 (cilium assembly; *p* = 9.38E-08) and ME13 (stem cell signature; *p* = 4.05E-41) fell outside the general classification. ME8 was enriched for both cell cycle (*p* = 7.49E-30) and immune-associated genes (*p* = 1.54E-29). Four of the top six largest modules ME2, ME3, ME5 and ME6 comprising the majority of lncRNAs (524/822) were associated with transcriptional regulation (Table 1), possibly reflecting the common role of lncRNAs in chromatin structure modification and control of PC gene expression [18]. The smaller modules were typically related to more specific signatures, including four modules associated with the immune system (ME8, ME9, ME11, ME15), three with the extracellular matrix (ME7, ME12, ME16), and three with neurological processes (ME5, ME10, ME14). No coherent functional signature could be established for the largest module (ME1) of 723 lncRNAs, and 288 lncRNAs were allocated to a pseudo-module (ME0) since their module membership could not be established. Overall, a putative pan-cancer functional association could be assigned to 822 lncRNAs by our approach.

**Table 1:** Module features

Module	Number of lncRNAs	Functional signature
ME0	288	None
ME1	723	None
ME2	317	Transcriptional regulation
ME3	128	Transcriptional regulation
ME4	56	Cilium assembly
ME5	46	Transcriptional regulation / Neurological
ME6	33	Transcriptional regulation
ME7	33	Extracellular
ME8	32	Immune / cell cycle
ME9	31	Immune / extracellular
ME10	30	Neurological
ME11	29	Immune
ME12	26	Extracellular
ME13	21	Stem cell
ME14	16	Neurological
ME15	12	Immune
ME16	12	Extracellular

#### Tissue type specificity

The functional themes of several modules reflected the cancer or normal tissue type specificity of their lncRNAs (Fig. 2c, Additional file 8: Table S5) [19]. As expected, neurological-associated ME5, ME10, and ME14 were highly specific to brain cancers, and all 21 lncRNAs of stem cell associated ME13 were detected in testicular germ cell tumours (TGCT), consistent with the notion that TGCT cells are derived from normal germ cells with distinct stem cell characteristics [20]. ME6 was also highly specific to TGCT, and whilst there was no significant association with a stem cell signature, it included the lncRNA *LINC-ROR*, which modulates reprogramming of fibroblasts to a pluripotent stem cell state [21]. Likewise, the enrichment of ME8 for immune processes such as lymphocyte activation (*p* = 1.54E-29) reflected its specificity for thymoma, and the origins of this cancer type in the thymus gland. Interestingly, ME8 was also associated with the cell cycle (*p* = 7.49E-30), which is emerging as a potential prognostic indicator in thymoma [22]. No disease bias was observed in transcriptional regulation-associated modules ME2 and ME3, immune-associated ME9 and ME15, and extracellular matrix-associated ME7, ME12 and ME16, suggesting that lncRNAs in these modules contribute to fundamental cellular processes common to most cancer types.

### Detailed characterisation of the extracellular-associated modules

Given their pan-cancer expression, and current poor understanding of the role of lncRNAs in extracellular processes, we were keen to dissect modules ME7, ME12 and ME16 further, and generate hypotheses on their potential function in supporting tumour progression.

#### FOS/JUND transcription factor binding site enrichment in ME7

**Fig. 3:**
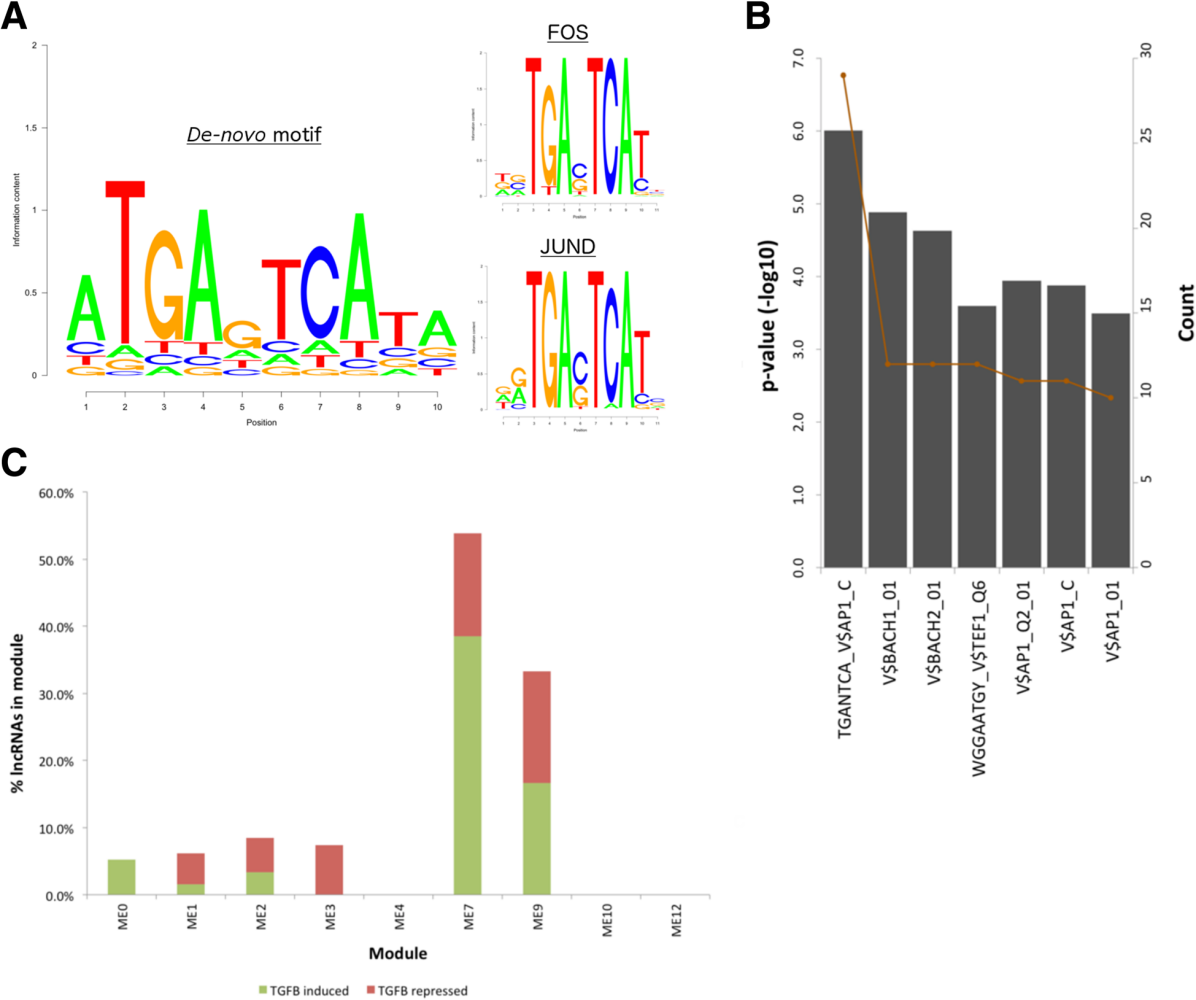
Evidence of c-Fos/c-Jun regulation in ME7. **a** Top-scoring *de-novo* motif in the region 1000 bp upstream of lncRNA transcription start site, and top two most similar JASPAR [23] transcription factor motifs. **b** Enrichment of AP1-like binding sites in 188 pro-module PC genes of ME7 according to [24]. **c** Proportion of lncRNAs in each module regulated by TGF-β and occupied by SMAD3 according to [24]. Only modules containing > 5 lncRNAs overlapping with those expressed in HSC myofibroblasts are shown

To establish whether lncRNAs in each of the extracellular modules share a common promoter, we performed a de novo search for sequence motifs in regions 1000 bp upstream of the lncRNA transcription start site (TSS). Whilst there was no evidence for transcription factor binding enrichment in ME12 and ME16, a top scoring motif achieving > 95% similarity with FOS and JUND transcription factor binding sites [23] was observed in 18/33 lncRNAs of ME7, (Fig. 3a; Additional file 9: Tables S6a-d). There was no evidence for enrichment of the FOS/JUND motif in the other 15 modules.

Underpinning this discovery, pro-module PC genes of ME7 were enriched for the binding site of activator protein-1 (AP-1) (29/188 pro-module PC genes; *p* = 9.80E-07; Fig. 3b; Additional file 10: Table S7), a transcription factor dimer of Jun and the Fos family of basic leucine zipper domain proteins, with FOS-like antigen 1 (*FOSL1*) achieving the highest PC-MA value of 0.036 (Additional file 6: Table S3). Moreover, strong binding of both c-Jun and c-Fos to the promoter region of ME7 lncRNA, *RP11-554I8.2* (also known as *LINP1*), has recently been confirmed in triple negative breast cancer cell lines [25].

Since c-Jun and c-Fos are known to co-operate with mothers against decapentaplegic homolog (SMAD) proteins to mediate transforming growth factor beta (TGFβ) signalling at AP-1 binding sites [26], we compared ME7 with two studies on SMAD targets [27, 28]. Firstly, overlap with [27] revealed 39% (13/33) of ME7 lncRNAs are expressed in human hepatic stellate cells (HSC) (Additional file 11: Table S8), representing the highest enrichment compared to the other modules. Of these, 53% (7/13) are potential targets for SMAD3 representing significant enrichment (*p* = 0.01 by hyper-geometric test), and either induced (39%; 5/13) or repressed (15%; 2/13) by TGFβ signalling (Fig. 3c, Additional file 11: Table S8). Similarly, comparison with [28] showed that the promoters of 13 of the top 20 ME7 pro-module genes could be occupied by either SMAD2 or SMAD3.

TGFβ induces epithelial-mesenchymal transition (EMT) in tumours via activation of SMAD proteins [29], which translocate into the nucleus and regulate transcription of TGFβ target genes [30]. Furthermore, since SMADs have low affinity for DNA, it is crucial they interact with cofactors such as AP-1 [31] to achieve target specificity. Exploring a potential link between ME7 and EMT induction via TGFβ signalling, we observed significant enrichment (*p* = 8.74E-20) for an EMT signature in pro-ME7 PC genes that included *SNAI2* (PC-MA = 0.025) and *TGFβ1* (PC-MA = 0.021). Pro-ME7 PC genes also included *HMGA2* (PC-MA = 0.024), a downstream effector of TGFβ during EMT [32], and *FOSL1*, whose protein product Fos-related antigen 1 (Fra-1) is implicated in EMT through modulation of TGFβ expression [24]. Taken together, our results indicate that lncRNAs of ME7 play a role in the induction of EMT via convergence of AP-1 and SMAD proteins at their promoters and regulation of TGFβ signalling.

#### Determination of the tumour stromal specificity of ME12 and ME16

**Fig. 4:**
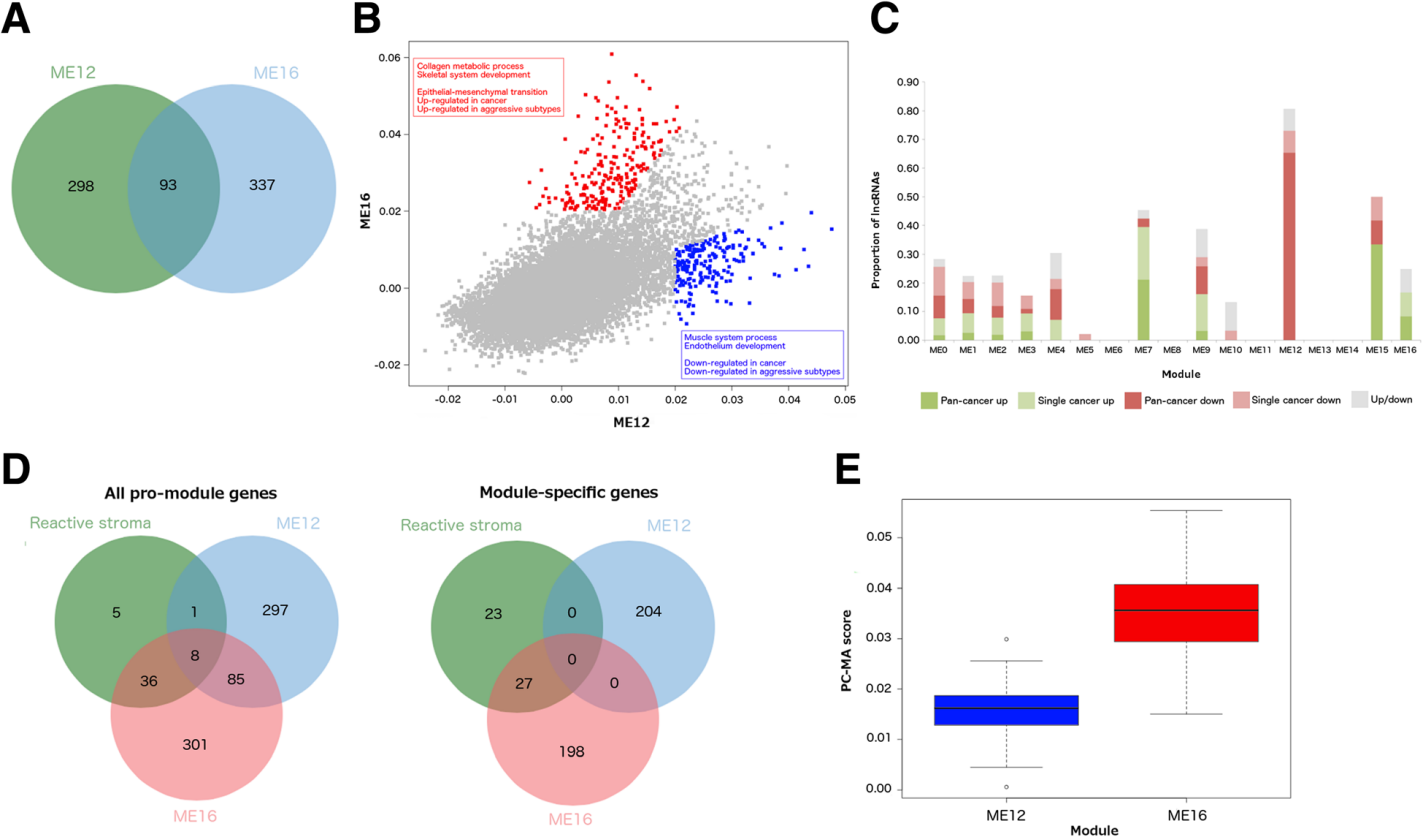
Differentiation of the extracellular-associated modules ME12 and ME16. **a** Venn diagram to show overlap of pro-module PC genes between ME12 and ME16. **b** Scatterplot of ME16 versus ME12 PC-MA values. ME16-specific PC genes are indicated in red, ME12 in blue with corresponding text highlighting signature enrichments in module-specific lists. **c** Proportion of lncRNAs potentially dysregulated in cancer within each module. **d** Venn diagrams showing overlap of all pro-module genes and module specific genes with a reactive stroma signature [33]. **e** Boxplot comparing PC-MA distribution across reactive stroma signature genes between ME12 and ME16

We noted that ME12 and ME16 shared a number of pro-module PC genes (Fig. 4a) and achieved significant correlation between their eigen-lncs (*r* = 0.57). In addition, both pro-module PC gene sets of ME12 and ME16 overlapped significantly (*p* < 0.05 by hyper-geometric test) with a stromal cell signature [34], incorporating 24% (32/136) and 35% (48/136) signature genes respectively. By contrast, no overlap was observed with ME7.

We explored the potential stromal specificity of ME12 and ME16 further by using a novel approach to generate a putative list of 300 stromal cell specific (SCS) lncRNAs frequently detected in stromal-containing clinical samples but not in pre-clinical models that consist almost exclusively of tumour cells (Additional file 12: Table S9a; see *Methods*). Both ME12 and ME16 contained an abundance of SCS lncRNAs, achieving 60% (15/25) and 53.6% (7/11) overlap respectively (Additional file 12: Table S9b). By contrast, only 1/27 (3.7%) lncRNAs in ME7 were classed as SCS. These results provide strong in silico evidence that expression of lncRNAs in ME12 and ME16 is specific to the tumour stroma.

#### Functional dissection of stromal-specific modules ME12 and ME16

To define more precise roles for the lncRNAs of ME12 and ME16, we identified 204 “ME12 specific” and 226 “ME16-specific” PC genes that achieved a PC-MA fold difference > 2.00 with the corresponding PC gene in ME16 and ME12 respectively (Fig. 4b; Additional file 13: Table S10a and S10b).

Comparison with signatures from MSigDB [35] revealed ME12-specific PC genes were consistently down-regulated in cancers including prostate (*p* = 1.79E-34) and colorectal (*p* = 3.10E-18), and advanced disease such as metastatic prostate cancer (*p* = 1.07E-12). They were also down-regulated in aggressive cancer subtypes such as luminal-B (*p* = 2.38E-17) and basal-like breast cancers (*p* = 9.12E-09). In contrast, ME16-specific PC genes were consistently associated with hallmarks of tumour progression such as EMT (*p* = 3.90E-67), and up-regulated in aggressive subtypes such as basal-like breast cancer (*p* = 1.00E-11).

Further evidence for under-expression of ME12 lncRNAs in cancer was provided by a systematic comparison of lncRNA expression between tumour and normal samples across 14 cancers types (see *Methods*). Each lncRNA was classified as “pan-cancer up” or “pan-cancer down” if differential expression was consistent across more than one cancer type, “single cancer up” or “single cancer down” if observed in a single cancer type, or “both” if the lncRNA was differentially expressed in both directions across different cancer types (Additional file 14: Table S11a and 11b). 73% (19/26) of lncRNAs in ME12 were classed as “pan-cancer down” or “single cancer down” (Fig. 4c, Additional file 14: Table S11c). These included the known tumour suppressor maternally expressed gene 3 (*MEG3*) [36], which was under-expressed in 4/14 cancers represented in our dataset. By contrast, only three lncRNAs in ME16 were classed as differentially expressed, and none as either “pan-cancer down” or “single cancer down”.

Interestingly, 39% (13/33) of lncRNAs in the third extracellular-associated module ME7 were classed as either “pan-cancer up” or “single cancer up”, with > 70% of these over-expressed in head and neck squamous cell carcinoma (HNSCC). This was concurrent with the strong association between ME7 and *FOSL1*, which is consistently over-expressed in HNSCC [37]. No evidence was observed of a relationship between tissue specificity (Fig. 2c) and up-regulation in specific cancer types (Additional file 14: Table S11). Of the modules showing tissue bias, only ME4 contained lncRNAs over-expressed in a single cancer type. ME4 had some tissue specificity to breast, however none of the four “single cancer up” lncRNAs were over-expressed in breast cancer.

#### Comparison of ME12 and ME16 with a reactive stroma signature

The above findings led us to compare ME12 and ME16 with a reactive stroma signature [38]. 44/50 genes (*p* < 6.46E-65 by hypergeometric test) in the signature overlapped with pro-module PC genes of ME16 compared to only 9/50 genes (*p* < 1.71E-6) with ME12 (Fig. 4d). These included fibroblast-activation protein (*FAP*; PC-MA = 0.06), an established cancer-associated fibroblast (CAF) marker, periostin (*POSTN*; PC-MA = 0.05) a gene implicated in metastasis [39], and members of the collagen family such as *COL5A2* (PC-MA = 0.05), *COL6A3* (PC-MA = 0.05), *COL10A1* (PC-MA = 0.04) and *COL6A1* (PC-MA = 0.04). Considering only module-specific genes, 27/50 genes (*p* < 3.17E-38) in the signature overlapped with ME16 but none with ME12 (Fig. 4d). Moreover, signature genes achieved significantly higher PC-MA values with ME16 than ME12 (*p* = 1.59E-20 by Student’s *t*-test; Fig. 4e, Additional file 15: Table S15). Taken together, our results strongly suggest that ME16 lncRNAs are markers of an activated stromal phenotype that promotes tumour progression, whereas ME12 lncRNA expression supports a tumour suppressive microenvironment.

#### Potential regulatory roles of lncRNAs in ME7, ME12 and ME16

We further assessed ME7, ME12 and ME16 in the context of lncRNA canonical interaction data collated by Chiu et al. [40]. In this study, tumour-type specific lncRNA interactions between effectors (transcription factors, micro-RNAs and RNA binding proteins) and their targets were inferred from eCLIP/ChIP-seq data, and transcription factor-promoter, micro-RNA-target and RNA binding protein-target predictions. This enabled us to assess our method against an alternative approach that does not solely rely on co-expression information.

Using breast cancer as an example, modules were first filtered for lncRNAs where interaction data were available in [40]. From those remaining, representative lncRNAs achieving the highest association score correlation with the eigen-lnc were then selected from ME16 (*RP11-863P13.3*), ME12 (*FENDRR*) and ME7 (*RP13-463 N16.6*), PC interactions with these lncRNAs were identified from [40]. In total, 433, 454 and 31 PC genes predicted to interact with ME16, ME12 and ME7 respectively were taken forward. In order to assess these interactions in the context of our method, we calculated mean PC-MA scores across each set of interacting PC genes and all modules (Table 2), with high PC-MA scores from the same module as the representative lncRNA indicating co-expression between lncRNA and its putative PC gene target.

**Table 2:** Mean PC-MA scores of lncRNA-interacting PC genes [40] associated with ME7, ME12 and ME16

Module	RP11-863P13.3 (ME16)		FENDRR (ME12)		RP13-463 N16.6 (ME7)	
	Mean PC-MA	Rank	Mean PC-MA	Rank	Mean PC-MA	Rank
ME0	−0.0028	17	−0.0013	16	−0.0015	13
ME1	−0.0024	16	−0.0019	17	−0.0005	8
ME2	0.0004	4	0.0006	3	0.0005	5
ME3	−0.001	13	−0.0009	15	0.0007	3
ME4	−0.0013	15	−0.0005	12	−0.0013	12
ME5	0.0005	3	−0.0002	7	0.0006	4
ME6	−0.0006	11	−0.0006	13	0.002	1
ME7	−0.0006	9	−0.0001	5	−0.0028	16
ME8	−0.0009	12	−0.0009	14	−0.0002	7
ME9	−0.0004	7	0.0001	4	−0.0006	9
ME10	−0.0013	14	−0.0004	9	−0.0006	10
ME11	−0.0006	8	−0.0003	8	0.0008	2
ME12	0.0016	2	0.002	1	−0.0017	14
ME13	−0.0006	10	−0.0005	11	−0.0009	11
ME14	−0.0001	5	−0.0001	6	0.0001	6
ME15	−0.0004	6	−0.0004	10	−0.0019	15
ME16	0.0027	1	0.0011	2	−0.0036	17

Interacting PC genes with *RP11-863P13.3* and *FENDRR* achieved the highest mean PC-MA scores in ME16 and ME12 respectively (Table 2), suggesting that lncRNAs of both these modules are co-expressed with their PC gene partners and typically activate their targets. Conversely, PC genes predicted to interact with *RP13-463 N16.6* achieved the second lowest mean PC-MA score in ME7 indicating negative expression correlation, and that lncRNAs of ME7 play an inhibitory role towards their targets. Interestingly, these included *JUND*, predicted to act as a transcription factor switch regulating multiple targets [40], supporting our earlier finding that lncRNAs of ME7 are enriched for *JUND* binding sites.

#### esiRNA knockdown of ME16 lncRNAs

Given the strong evidence for their stromal cell specificity, and association with activated stroma, we took forward two lncRNAs of ME16 (*AC093850.2* and *RP11-626H12.2*) to experimentally assess their role in the tumour microenvironment, alongside a lncRNA not associated with this module (*RP1-122P22.2* from ME2) and a non-targeting esiRNA (Evf-2) as negative controls. The nearest upstream neighbour of *AC093850.2* is fibronectin (*FN1*), thus providing a potential example of a *cis*-relationship between a known fibroblast marker and lncRNA. *AC093850.2* is also predicted as interacting with *FN1* in breast cancer [40], acting as a microRNA/RNA binding protein decoy. To our knowledge, there is no evidence that the protein coding neighbours of *RP11-626H12.2* play a direct role in CAF activation.

**Fig. 5:**
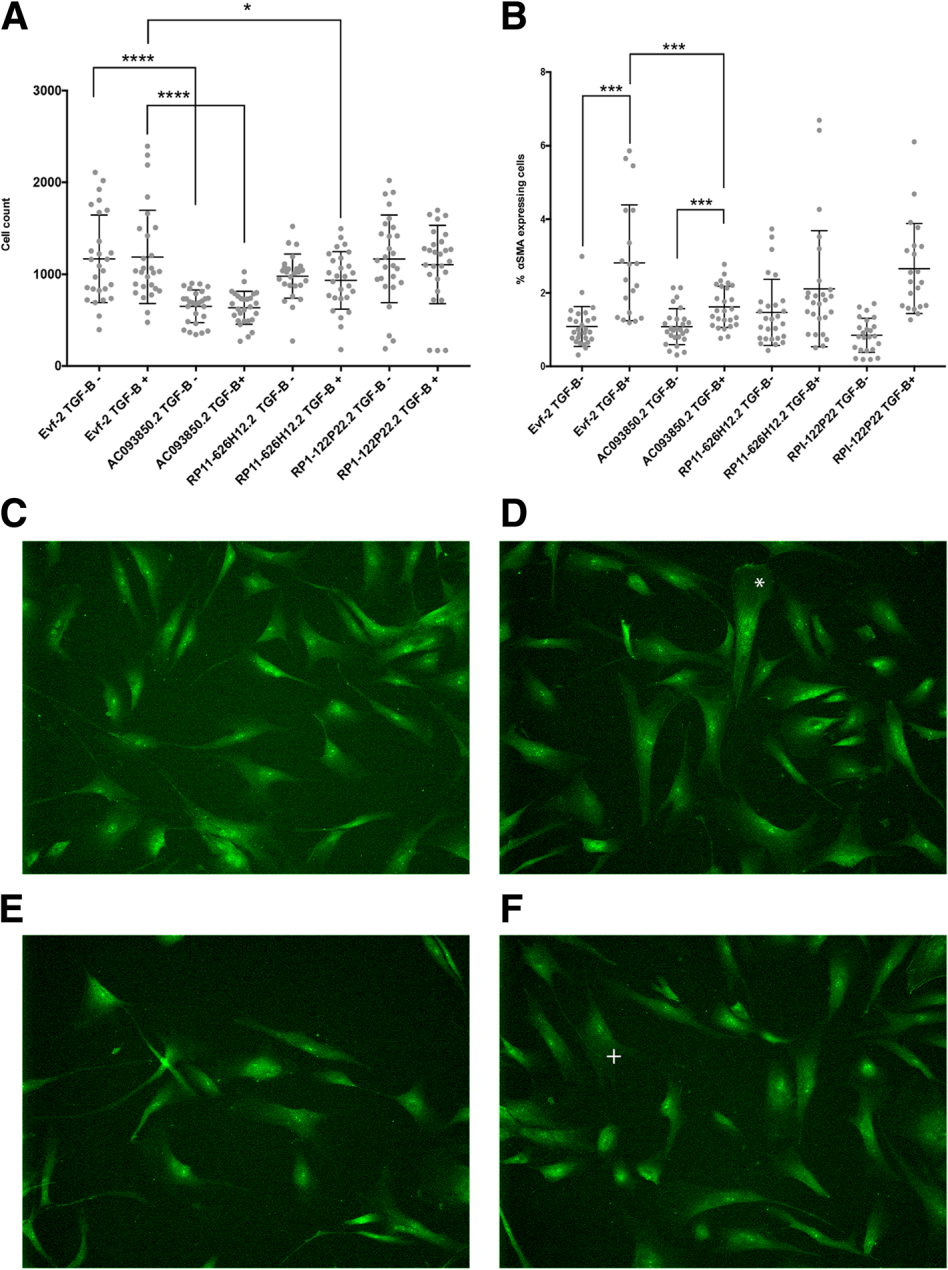
A candidate lncRNA is able to attenuate TGF-β1-induced fibroblast differentiation. Human primary fibroblasts were transfected with esiRNA targeting two candidate lncRNAs (*AC093850.2* and *RP11-626H12.2*), a transcript not expressed in human cells (Evf-2) and a lncRNA from a different module not predicted to influence fibroblast differentiation (*RP1-122P22.2*). Each experiment consisted of nine technical and three biological replicates. **a** Cells were dispensed into 384 well plates, reverse transfected with esiRNAs, incubated for 2 days knock-down and then stimulated or not with TGF-β1 for 24 h. Images were acquired using a MetaXpress Micro × 2 objective and cells identified using the nuclei stain Hoechst, and segmented using MetaXpress software. Data processed in Excel and Prism7. **b** The protocol was identical to that of A, but the cell were stained with αSMA antibody after fixation, and imaged using the × 20 objective. Positive CAFs were identified on the formation of de novo αSMA-positive stress fibres and morphological changes using MetaXpress Custom Module Editor. **p < 0.05, ***p < 0.01, ****p < 0.001. Only comparisons between groups reaching statistical significance are indicated. **c** Microscope images of unstimulated, control Evf-2 knock-down cells. **d** TGF-β1-stimulated, control Evf-2 knock-down cells. The white * indicates a transformed CAF with both morphological and αSMA positive fibres. **e** Unstimulated, *AC093850.2* knock-down cells. **f** TGF-β1-stimulated, *AC093850.2* knock-down cells. The white + indicates a cell counted as a CAF with a partial transformation

We used an established experimental model of CAF differentiation [41] that uses TGF-β1 to activate human primary fibroblasts, assessed as induction of alpha-smooth muscle actin (αSMA), a commonly used CAF marker (Fig. 5a). CAFs are the major cell type in the tumour microenvironment and are known to play a role in the invasion and metastasis of tumour cells [42, 43]. There is strong evidence showing association between CAFs and poor prognosis in several types of cancers [44]. Figure 5a shows that the knock-down of *AC093830.2* has an effect on cell number, but is not completely required for cell viability. We observed that TGF-β1-mediated activation of fibroblasts (as assessed by the number of cells harbouring αSMA-positive stress fibres determined by immunofluorescent labelling and high content microscopy) is impaired when expression of both candidate lncRNAs, but not lncRNA from different functional modules, is reduced in human fibroblasts using specific esiRNAs (Fig. 5b). This reduction in TGF-β1-mediated stress-fibre formation reached statistical significance for one of the lncRNAs, *AC093830.2*, when compared to the response in the presence of esiRNA targeting a gene not expressed in these cells (Evf-2). The magnitude of reduction observed is modest; this may be a result of relatively low levels of activation by TGF-β1 in this system, in which basal αSMA is readily detected. Future studies using more sophisticated 3D in vitro models, more accurately recapitulating the quiescent in vivo conditions in which basal αSMA is lower, are likely to reveal more pronounced effects of perturbing responses to TGF-β1 stimulation and associated physiological importance.

In comparison to control (Fig. 5c and d) and unstimulated *AC093850.2* knock-down (Fig. 5e), images of TGF-β1 activated fibroblasts knocked-down with *AC093830.2* RNAi show that the cells are morphologically different to activated wild-type CAFs, and a few cells still show some transformation into the TGF-β1 induced phenotype (Fig. 5f, white cross). These results suggest that *AC093850.2* is functionally linked with the differentiation of fibroblasts to a CAF phenotype, and may act in a redundant manner.

Further support for the association of ME16 with the CAF phenotype was provided by analysis of gene expression data from a separate study using the same method of induction of a CAF phenotype [45]. A comparison showed that 20/64 genes over-expressed (log_2_FC > 1.50, *p* < 1.00E-04) in response to TGF-β1 treatment of HFFF2 fibroblasts are also members of the pro-module gene set of ME16, representing significant overlap (*p* = 1.47E-14 by hypergeometric test).

## Conclusion

In this study, we present the most comprehensive examination of the pan-cancer lncRNA expression landscape to date. A key contribution is the development of a novel approach to integrate transcriptome data across multiple cancers, allowing us to generate lncRNA-PC networks and de-convolute lncRNAs into a small number of functionally coherent modules. By doing so, we provide some important insights and hypotheses into the role of lncRNAs in cancer. Principally, lncRNAs can be grouped into just four functional themes based on their associations with PC genes: immune, extracellular, transcription regulation, and neurological.

Whilst a number of modules are clearly driven by the tissue specificity of their lncRNAs, several pan-cancer modules are identified, of which three may represent distinct lncRNA networks associated with extracellular processes that regulate key events in tumour progression. Two of these modules are stromal specific, corresponding to a 26-lncRNA signature associated with a tumour suppressive microenvironment, and 12 lncRNAs with a potential role in cancer fibroblast activation leading to poor prognosis. The third module consists of a tumour-derived signature of 33 lncRNAs that may play a role in inducing EMT through modulation of TGFβ signalling. Adding confidence to our approach, our findings complemented a previous study that used an alternative method for assessing lncRNA-PC gene associations. Furthermore, the potential functional regulatory roles of two members of the putative lncRNA CAF signature were validated by experimental modulation in fibroblasts. Interestingly, whilst reduction in TGF-β1-mediated stress-fibre formation was observed for both lncRNAs, it reached statistical significance only for *AC093850.2* (also known as *LINC01614*). The nearest upstream neighbour of *AC093850.2* is fibronectin, a key component of CAF-derived ECM known to influence matrix remodelling associated with metastasis [46]. Therefore, our findings could indicate a lncRNA-mediated control mechanism of fibroblast differentiation via *cis*-regulation of fibronectin by *AC093850.2*.

Since reference to modules alone may mask subtle functional differences that exist between lncRNAs, we encourage researchers to explore the individual lncRNA PC-MA profiles provided as supplementary data (https://figshare.com/s/753cc0df15197b0b9572). Together with the modules, they provide a unique, global compendium from which to generate novel hypotheses and motivate detailed functional studies on lncRNA roles in cancer.

## Methods

### TCGA RNA-Seq data processing

Raw FASTQ sequence files for each solid tumour represented in TCGA were downloaded from the Cancer Genomics Hub (CGHub; [47]), and reads aligned to the human (GRCh38) genome using StarAlign [48] with no more than three mismatches and only uniquely mapped reads allowed. Reads whose ratio of mismatches to mapped length was greater than 0.10 were also discarded. All other parameters were set to their defaults for unstranded alignment. To reduce possible biases introduced by variable total read counts between samples, tumours achieving < 20,000,000 mapped reads were removed. The expression level, based on Fragments Per Kilobase per Million fragments mapped (FPKM), of each gene present in the human (GRCh38) GENCODEv22 annotation file was estimated using Cufflinks with library type defined as “fr-unstranded” and all other parameters set to defaults [49]. Expression values were then batch normalized using COMBAT [50] where appropriate. Only genes annotated as “lincRNA” or “protein_coding” were considered. LncRNAs overlapping PC genes, such as antisense transcripts, were ignored. Since expression across transcripts less than the average RNA fragment length can be over-estimated, genes whose largest transcript was less that 400 bp were also ignored. We also removed lncRNA and PC genes that failed to achieve sufficient expression signal across at least one cancer type. Specifically, the sum of the mean and standard deviation of FPKMs across each cancer type for each lncRNA and PC gene were calculated. If the maximum value of this sum across all cancer types fell below 1.00, then the gene was discarded. The resulting gene-by-sample matrix consisted 17,088 PC genes and 2098 lncRNAs. Note that a poly-A selection protocol was used for TCGA RNA-Seq, and so lncRNAs are restricted to these species. Sequencing data for all TCGA cancer types used in this study were processed using the same procedure. The number of tumours across each cancer type is given in Additional file 4: Table S1.

### Pan-cancer estimation of the correlation between each lncRNA and PC gene

Visual inspection of the data indicates that a three-component mixture distribution is an appropriate representation. The first two densities can be seen to decay exponentially away from the x and y axes and the third distribution looks bivariate Gaussian (Additional file 2: Figure S2). We use the expectation maximisation (EM) algorithm to estimate the parameters of our statistical mixture model. Since we are specifically interested in the correlation coefficient of the bivariate Gaussian density, we estimate the separate parameters of the bivariate Gaussian covariance matrix rather than the whole covariance matrix itself. To exploit the convenience of using sufficient statistics for the parameters, we ensure that the mixture density is in the exponential family. Data across 32 cancer types (indexed by *c*) is used in the maximum likelihood estimation. The three-component mixture density likelihood over the 32 cancer types is:

$$ \prod \limits_{c=1}^{32}\prod \limits_{i=1}^{n_c}\left[{w}_{c1}{f}_1\left({x}_{ci1}\right)+{w}_{c2}{f}_2\left({x}_{ci2}\right)+{w}_{c3}{f}_3\left({x}_{ci1,}{x}_{ci2}\right)\right] $$

where *w*_*cj*_ is the weight for component *j* in cancer type c ($\mathrm{such}\ \mathrm{that}\ \sum \limits_{j=1}^3{w}_{cj}$ =1 for all cancer types), *n*_*c*_ is the number of samples in cancer type *c*, *x*_*ci*1_ is the *i*th lncRNA gene expression value and *x*_*ci*2_ is the *i*th PC gene expression value in cancer type *c.* The three mixture components are

$$ {f}_1\left({x}_{ci1,}{x}_{ci2}\right)=\frac{6}{\varGamma \left(1/6\right)-\gamma \left(1/6,1\right)}\exp \left(-{\left({x}_{ci1}+0.86\right)}^6\right) $$

$$ {f}_2\left({x}_{ci1,}{x}_{ci2}\right)=\frac{4}{\varGamma \left(1/4\right)-\gamma \left(1/4,1\right)}\exp \left(-{\left({x}_{ci2}+0.86\right)}^4\right) $$

$$ {f}_3\left({x}_{ci1,}{x}_{ci2}\right)\propto \prod \limits_{i=1}^{n_c}\exp \left(\frac{1}{-2\left(1-{\rho}^2\right)}\left[{\left(\frac{x_{ci1}-{\mu}_{c1}}{\sigma_1}\right)}^2+{\left(\frac{x_{ci2}-{\mu}_{c2}}{\sigma_2}\right)}^2-2\rho \left(\frac{x_{ci1}-{\mu}_{c1}}{\sigma_1}\right)\left(\frac{x_{ci2}-{\mu}_{c2}}{\sigma_2}\right)\right]\right) $$

where *Γ* is the standard gamma function and *γ* the lower incomplete gamma function. In order to fit this into the exponential family we assume that the lncRNA and PC gene expression variances for each of the cancer types are identical and defined as *σ*_1_^2^ and *σ*_2_^2^. The lncRNA and PC gene expression expectations (*μ*_*c*1_ and *μ*_*c*2_) are however allowed to vary for each of the cancer types. The correlation coefficient *ρ* is the parameter of interest.

We use the EM algorithm with updates derived by equating expectations in the usual way. Let ${w}_{cm}^{\ast }$ represent the current value of the parameter estimates of the *m*^th^ mixture weight (*m* = 1, 2, 3) in cancer type *c*. Let *Θ*^∗^ represent the current value of all the remaining parameters, let *i* represent the sample number in cancer type *c* (1 ≤ *i* ≤ *n*_*c*_) and let

$$ {p}_{ci m}=\frac{w_{cm}^{\ast }{f}_m\left({x}_{ci1,}{x}_{ci2}|{\varTheta}^{\ast}\right)}{\sum \limits_{j=1}^3{w}_{cj}^{\ast }{f}_j\left({x}_{ci1,}{x}_{ci2}|{\varTheta}^{\ast}\right)}. $$

Then the EM updates are as follows:

$$ {w}_{cm}=\sum \limits_{i=1}^{n_c}{p}_{cim}/{n}_c $$

$$ {\mu}_{cm}=\sum \limits_{i=1}^{n_c}{x}_{ci1}{p}_{ci m}/\left({n}_c{w}_{c3}\right) $$

$$ {\sigma}_1^2=\frac{\sum \limits_{c=1}^{32}\left\{\sum \limits_{i=1}^{n_c}{x}_{ci1}^2{p}_{ci3}-{n}_c{w}_{c3}{\mu}_{c1}^2\right\}}{\sum \limits_{c=1}^{32}{n}_c{w}_{c3}} $$

$$ {\sigma}_2^2=\frac{\sum \limits_{c=1}^{32}\left\{\sum \limits_{i=1}^{n_c}{x}_{ci2}^2{p}_{ci3}-{n}_c{w}_{c3}{\mu}_{c2}^2\right\}}{\sum \limits_{c=1}^{32}{n}_c{w}_{c3}} $$

$$ \hat{\rho}=\frac{\sum \limits_{c=1}^{32}\left\{\sum \limits_{i=1}^{n_c}{x}_{ci1}{x}_{ci2}{p}_{ci3}-{n}_c{w}_{c3}{\mu}_{c1}{\mu}_{c2}\right\}}{\sigma_1^2{\sigma}_2^2\sum \limits_{c=1}^{32}{n}_c{w}_{c3}} $$

### Accounting for the uncertainty of the estimated pan-cancer correlation

Here $\hat{\rho}$ is a pan-cancer measure of correlation between lncRNA and PC gene. For each correlation estimate, we calculate the standard error of the estimate ($SE\left(\hat{\rho}\right)$) by bootstrapping with 100 bootstrap samples. This enables us to use a measure of the pan-cancer correlation that takes the uncertainty of the estimate into account, namely $\hat{\rho}/ SE\left(\hat{\rho}\right),$ which we refer to as the MCA score. Where lncRNA or PC gene expression signal is insufficient to calculate a correlation estimate, the cancer type is not considered further for this combination. In a significant number of cases, low expression of the lncRNA means the correlation cannot be estimated, and thus failure to calculate an MCA score for a specific PC gene. Where this occurs for over 50% of the PC genes, the lncRNA is not considered further, resulting in removal of a further 265 lncRNAs. Overall, 1833 lncRNAs have an MCA score for more than 50% of the 17,088 PC genes.

### Weighted correlation network analysis (WGCNA)

To perform WGCNA [17], the R package “*WGCNA*” was applied as follows. First, a weighted lncRNA MCA score correlation network was constructed from the 1833 lncRNA by 17,088 PC gene MCA score matrix using a soft thresholding power of 7 to which the MCA score correlation was raised to calculate adjacency. To aid choice of soft thresholding power we used the “pickSoftThreshold” WGCNA function with candidate powers 1–10, 12, 14, 16, 18 and 20. The power 7 was the lowest power for which the scale-free topology fit index reached 0.95 (Additional file 3: Figure S3A, resulting in a network with mean connectivity of 5.94 (Additional file 3: Figure S3B). Modules were then identified by average linkage hierarchical clustering of lncRNAs, and modules identified in the resulting dendrogram by the Dynamic Hybrid tree cut using signed topographical overlap matrix (TOM) and network types, a minimum module size of five, and a threshold for merging high correlated modules of 0.25. All other parameters were set to their default values.

### Signature enrichment analysis

Functional, cell type, transcription factor and disease type enrichment analyses were performed on each set of pro- and anti-module PC genes using Toppgene [33]. Significant enrichments were defined as those achieving False Discovery Rate less than 0.05 and signature overlap greater than two genes.

### Differential expression between tumour and normal samples

RNA-Seq raw FASTQ sequence files for TCGA matched normal samples across 24 cancer types were downloaded from CGHub [47], and gene expression estimates derived using the same procedure as for the tumour samples. Reads aligned to the human (GRCh38) genome using StarAlign [48] with no more than three mismatches and only uniquely mapped reads allowed. Reads whose ratio of mismatches to mapped length was greater than 0.10 were also discarded. All other parameters were set to their defaults for unstranded alignment. FPKM expression estimates of each gene present in the human (GRCh38) GENCODEv22 annotation file were calculated using Cufflinks with library type defined as “fr-unstranded” and all other parameters set to defaults [49]. Expression values were then batch normalized using COMBAT [50] where appropriate. 10 cancer types comprised of < 10 samples after filtering so were removed from further analyses (Additional file 4: Table S1). Differentially expressed lncRNAs (|log_2_FC| > 1.0 and *p* < 0.0001) between tumour and normal samples representing each of the remaining 14 cancer types were detected using the Student’s *t*-test on FPKM expression estimates.

### De novo transcription factor motif discovery

Nucleotide sequences 1000 bp upstream of each lncRNA were downloaded from Ensembl version 84 [51], and grouped according to module membership. Conserved motifs within these sequences from ME4 and ME5, and ME7-ME16 were then determined by a Weeder 2.0 [52] de novo search with default parameters. Modules without a coherent functional/cell type signature (ME1) or associated with transcriptional regulation only (ME2, ME3, ME6) were ignored. Motif matrices achieving scores > 2.0 were then assessed for similarity with transcription factor binding sites contained within the JASPAR database using the JASPAR matrix alignment tool [23]. De novo matrices achieving > 95% with a JASPAR matrix were deemed significant. Motifs associated with lncRNAs of ME13 were manually inspected using the Repeat Masker (http://www.repeatmasker.org) track on the University of California Santa Cruz (UCSC) Genome Browser [53].

### A novel approach to identify stromal cell specific lncRNAs

To further establish the stromal cell specificity of lncRNAs in ME12 and ME16, we used a novel approach to compare their expression in sample types that consist exclusively of tumour cells (stroma^low^) with fresh frozen TCGA patient samples that naturally contain a mixed population of tumour and stromal cells (stroma^high^). We reasoned that lncRNAs detected in stroma^high^ but not in stroma^low^ samples were likely stromal cell specific (for this purpose, immune cells are included in the definition of “stroma”). To represent stroma^low^ samples, we used 828 cell lines from the Cancer Cell Line Encyclopaedia (CCLE; Additional file 16: Table S13) [54], and 57 PDX models [55], in which tumour had been separated from stroma using an in silico species-specific mapping strategy [55, 56]. As expected, both stroma^low^ cohorts achieved a mean estimated tumour cell content of 99% ± 1%, compared to patient samples from TCGA where only 8/32 cancer types achieved median tumour cell content> 90% (Additional file 15: Table S12).

BAM files consisting of reads mapped to the human (GRCh37) genome were downloaded from the CGHub for the 828 cell lines representing 19 solid cancer types (Additional file 16: Table S13). Only cancer types represented in the TCGA dataset were considered. FPKM values for each gene present in the human (GRCh38) GENCODEv19 annotation file were calculated as before using Cufflinks with library type defined as “fr-unstranded”.

RNA-Seq data for the 57 PDX models representing eight cancer types (25 lung, 12 breast, 7 colorectal, 3 endometrial, 6 ovarian, 2 pancreatic, 1 ampullary and 1 leukaemia) were downloaded from ArrayExpress (accession number: E-MTAB-3980), and tumour and stromal expression separated according to [55]. Note that the tumour components of 22/69 PDX models in the original dataset showed evidence of patient stroma retention (mRNA expression of CAF markers *FAP* or *CSPG4* log_2_ FPKM> 2.0) so were ignored [55].

For the 1540 lncRNAs common to TCGA, CCLE and PDX datasets, we counted the number of tumour types in which the lncRNA was undetected in cell lines but detected in patient tumours (*x*), and the number of tumour types in which lncRNA was detected in patients regardless of cell line expression (*y*). Here, “detected” in patient tumours was defined as median FPKM> 1.00 across the cancer type, and “undetected” in cell lines defined as median FPKM< 0.50. 496 lncRNAs achieved *x*/*y* ≥ 0.50 and *x* > 1, or *x*/*y* = 1.00 and *x* = 1, and therefore classed as undetected in cell lines and detected in patient tumours (set A). 768 lncRNAs were classed as undetected in our PDX cohort, achieving a median read count across the 57 models of zero (set B). 300 lncRNAs formed the union of sets A and B, and were therefore classed as stromal cell specific (SCS) achieving expression in patient tumours but low or undetectable expression in either cell lines or PDX models. SCS lncRNAs included *MEG3*, one of the few lncRNAs established as preferentially expressed in tumour stroma [57], thus adding confidence to our approach.

### esiRNA knockdown

esiRNAs were prepared as described in [58] using DEQOR [59] and primer3 [60] for optimized design of the template. An in vitro transcription kit (Thermo) was used to generate the dsRNA according to manufacturer’s instructions, followed by SureCut RNase III (NEB) digestion. After testing for complete digestion prior to use by agarose gel electrophoresis, esiRNAs were transfected into human primary fibroblasts at 5 ng per well in a total volume of 25 μl. After 48 h, TGF- β1 (R and D Systems; 5 ng/ml) was added in serum-free medium. After a further 24 h, fibroblasts were fixed in formaldehyde and monitored for αSMA induction using high content microscopy and αSMA immunofluorescence, detected using a FITC-conjugated anti- αSMA monoclonal antibody (Sigma).

## Additional files

Additional file 1: **Figure S1.** Heatmap of eigen-lnc adjacencies. Each row and column corresponds to one eigen-lnc. Within the heatmap, red indicates high adjacency (positive correlation) and green low adjacency (negative correlation) as shown by the colour legend. (TIF 1166 kb) (TIF 1166 kb)

Additional file 2: **Figure S2.** Typical three-component mixture distribution observed between PC and lncRNA gene expression. The plot shows typical patterns of PC and lncRNA gene expression. Each point is a sample. Three clusters are visible: two are along the x and y axes, and the third is centred away from the axes. We model these data using a three-component mixture distribution. Two of the distributions run along and close to the x and y axes, and are designed to represent the data points near the axes. The third component is a bivariate Gaussian distribution (elliptical/circular in shape) designed to represent the points some distance way from the axes. Our focus is in estimating the correlation in the bivariate Gaussian component, but we use a mixture distribution to allow for the observations near the axes. Failure to do so would result in biased estimates of the correlation. (TIF 5061 kb) (TIF 5061 kb)

Additional file 3: **Figure S3.** Analysis of lncRNA-PC MCA score network topology for various soft-thresholding powers. A. The scale-free fit index (y-axis) as a function of the soft-thresholding power (x-axis). B. mean connectivity (degree, y-axis) as a function of the soft-thresholding power (x-axis). (TIF 2645 kb) (TIF 2645 kb)

Additional file 4: **Table S1.** Number of TCGA patients contributing to this study across 32 cancer types. (XLSX 42 kb)

Additional file 5: **Table S2.** Module assignment and correlation of lncRNA association score profiles with the eigen-lncs. (XLSX 589 kb)

Additional file 6: **Table S3.** Eigen-lnc coefficients (PC-MA scores) contributed by each protein coding gene. (XLSX 4910 kb)

Additional file 7: **Table S4.** ToppGene functional enrichment in pro-module protein coding genes. (XLSX 200 kb)

Additional file 8: **Table S5.** Module disease specificity. (XLSX 57 kb)

Additional file 9: **Table S6.** Evidence for FOS/JUN transcription factor binding sites in lncRNA promoters of module 7. (a) Weeder motif scores. (b) Frequency matrix associated with top scoring motif (ATGAGTCATA). (c) Presence of top-scoring motif in ME7 lncRNAs. (d) Top 6 JASPAR database matches with top matrix hit (human-derived motifs only). (XLSX 56 kb)

Additional file 10: **Table S7.** Enrichment of AP1 transcription factor binding sites in protein coding genes achieving PC-MA in module 7. (XLSX 31 kb)

Additional file 11: **Table S8.** Number and percentage lncRNAs in each module with ChipSeq evidence of SMAD3 occupancy. (XLSX 43 kb)

Additional file 12: **Table S9.** LncRNA detection in pre-clinical tumour models. (a) Assessment of expression levels of each lncRNA in cell line and PDX tumour models. (b) Number and proportion of lncRNAs detected in cell lines/PDX models in each module. (XLSX 166 kb)

Additional file 13: **Table S10.** Module-specific gene lists of extracellular-associated modules. (a) ME16-specific. (b) ME12- specific. (XLSX 70 kb)

Additional file 14: **Table S11.** Frequency of module-associated lncRNA dysregulation in cancer. (a) LncRNAs differential expressed in each cancer. (b) LncRNAs differentially expressed in at least one cancer type and their dysregulation classification. (c) Number and proportion of each dysregulation class in each module. (XLSX 78 kb)

Additional file 15: **Table S12.** PC-MA scores of genes in reactive stroma signature. (XLSX 58 kb)

Additional file 16: **Table S13.** Number of CCLE cell lines contributing to this study across 19 cancer types. (XLSX 21 kb)

## Data Availability

All data generated or analysed during this study are included in this published article and its supplementary information files, or available in the figshare repository https://figshare.com/s/753cc0df15197b0b9572.

## Abbreviations

CCLE: Cancer Cell Line Encyclopaedia
EM: Expectation Maximisation
FPKM: Fragments Per Kilobase per Million fragments mapped
HNSCC: Head and neck squamous cell carcinoma
MA: Module association
MCA: Multi-cancer association
ME: Module eigen-gene
PC: Protein coding
PDX: Patient derived xenograft
SCS: Stromal cell specific
TCGA: The Cancer Genome Atlas
TGCT: Testicular germ cell tumours
UCSC: University of California Santa Cruz
WGCNA: Weighted correlation network analysis
