# Supplementary figures and images for: Comprehensive functional profiling of long non-coding RNAs through a novel pan-cancer integration approach and modular analysis of their protein-coding gene association networks
Source: BMC Genomics. 2019 Jun 3;20:454. doi: 10.1186/s12864-019-5850-7 (PMC6547491; doi:10.1186/s12864-019-5850-7)

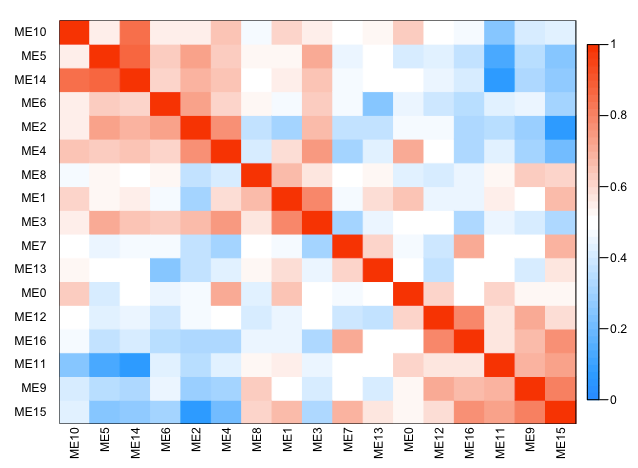

Supplement: Supplementary file 1 — Figure S1. Heatmap of eigen-lnc adjacencies. Each row and column corresponds to one eigen-lnc. Within the heatmap, red indicates high adjacency (positive correlation) and green low adjacency (negative correlation) as shown by the colour legend. (TIF 1166 kb) (TIF 1166 kb) [file 12864_2019_5850_MOESM1_ESM.tif]

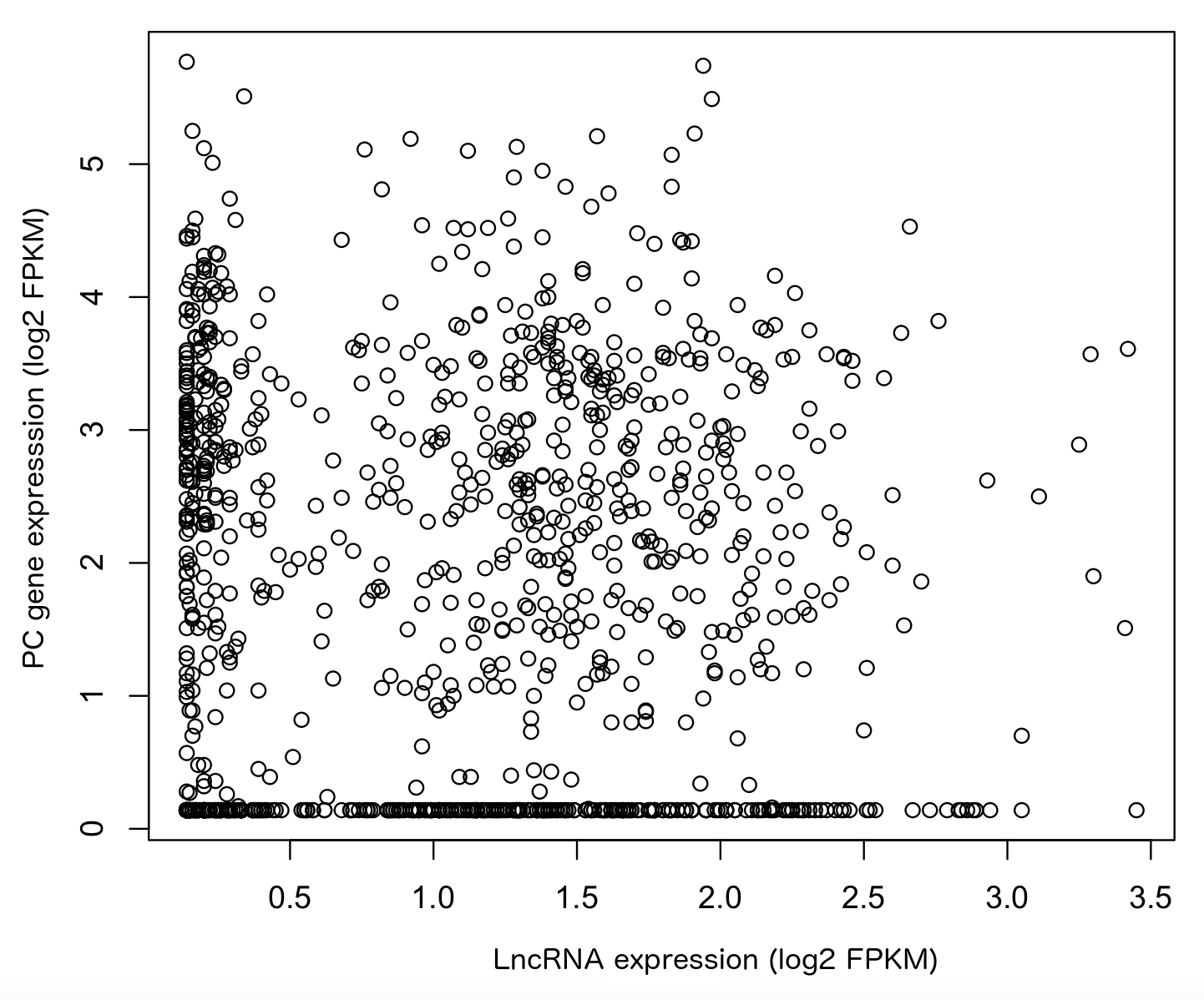

Supplement: Supplementary file 2 — Figure S2. Typical three-component mixture distribution observed between PC and lncRNA gene expression. The plot shows typical patterns of PC and lncRNA gene expression. Each point is a sample. Three clusters are visible: two are along the x and y axes, and the third is centred away from the axes. We model these data using a three-component mixture distribution. Two of the distributions run along and close to the x and y axes, and are designed to represent the data points near the axes. The third component is a bivariate Gaussian distribution (elliptical/circular in shape) designed to represent the points some distance way from the axes. Our focus is in estimating the correlation in the bivariate Gaussian component, but we use a mixture distribution to allow for the observations near the axes. Failure to do so would result in biased estimates of the correlation. (TIF 5061 kb) (TIF 5061 kb) [file 12864_2019_5850_MOESM2_ESM.tif]

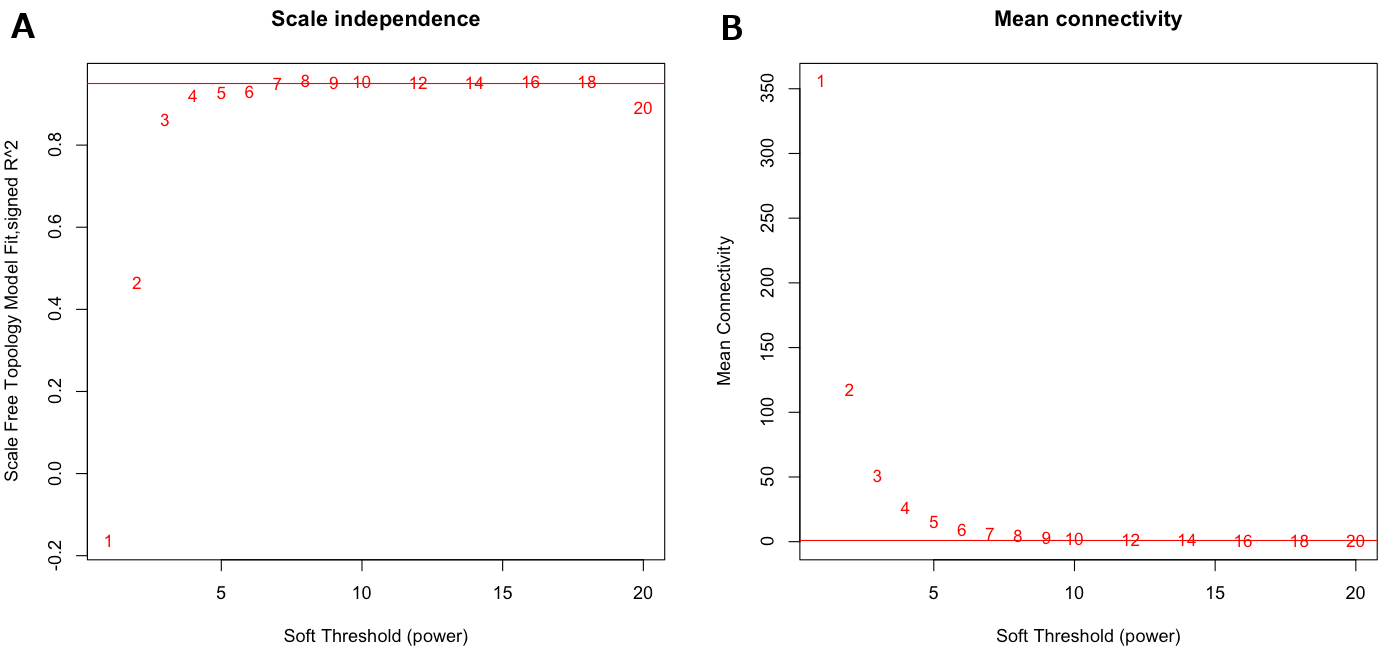

Supplement: Supplementary file 3 — Figure S3. Analysis of lncRNA-PC MCA score network topology for various soft-thresholding powers. A. The scale-free fit index (y-axis) as a function of the soft-thresholding power (x-axis). B. mean connectivity (degree, y-axis) as a function of the soft-thresholding power (x-axis). (TIF 2645 kb) (TIF 2645 kb) [file 12864_2019_5850_MOESM3_ESM.tif]
